# Evaluating the intersection of a regional wildlife connectivity network with highways

**DOI:** 10.1186/2051-3933-1-12

**Published:** 2013-11-22

**Authors:** Samuel A Cushman, Jesse S Lewis, Erin L Landguth

**Affiliations:** U.S. Forest Service, Rocky Mountain Research Station, 2500 S Pine Knoll Dr, Flagstaff, AZ 86001 USA; Graduate Degree Program in Ecology, Department of Fish, Wildlife, and Conservation Biology, Colorado State University, Fort Collins, CO 80523 USA; Division of Biological Sciences, University of Montana, Missoula, MT 59812 USA

**Keywords:** American black bear, Connectivity, Corridor, Crossing structures, Highways, Northern Rocky Mountains, Road effects, UNICOR

## Abstract

**Background:**

Reliable predictions of regional-scale population connectivity are needed to prioritize conservation actions. However, there have been few examples of regional connectivity models that are empirically derived and validated. The central goals of this paper were to (1) evaluate the effectiveness of factorial least cost path corridor mapping on an empirical resistance surface in reflecting the frequency of highway crossings by American black bear, (2) predict the location and predicted intensity of use of movement corridors for American black bear, and (3) identify where these corridors cross major highways and rank the intensity of these crossings.

**Results:**

We used factorial least cost path modeling coupled with resistant kernel analysis to predict a network of movement corridors across a 30.2 million hectare analysis area in Montana and Idaho, USA. Factorial least cost path corridor mapping was associated with the locations of actual bear highway crossings. We identified corridor-highway intersections and ranked these based on corridor strength. We found that a major wildlife crossing overpass structure was located close to one of the most intense predicted corridors, and that the vast majority of the predicted corridor network was “protected” under federal management. However, narrow, linear corridors connecting the Greater Yellowstone Ecosystem to the rest of the analysis area had limited protection by federal ownership, making these additionally vulnerable to habitat loss and fragmentation.

**Conclusions:**

Factorial least cost path modeling coupled with resistant kernel analysis provides detailed, synoptic information about connectivity across populations that vary in distribution and density in complex landscapes. Specifically, our results could be used to quantify the structure of the connectivity network, identify critical linkage nodes and core areas, map potential barriers and fracture zones, and prioritize locations for mitigation, restoration and conservation actions.

**Electronic supplementary material:**

The online version of this article (doi:10.1186/2051-3933-1-12) contains supplementary material, which is available to authorized users.

## Background

Population connectivity is important for maintaining genetic diversity and demographic exchange in regional populations [1–3], and for species to shift geographic ranges in response to climate change [4]. As a result, evaluating population connectivity and mapping linkage zones are of high importance in the face of increasing habitat loss and fragmentation and the threat of climate change [5–7]. Roads are unprecedented features in the ecological history of landscapes and potentially affect many ecological processes [8–10]. For example, road density has strong negative relationships to habitat quality and is one of the most powerful predictors of occurrence for many large mammals, such as grizzly bears [11, 12], elk [13–15], and wolves [16–18]. In addition, carnivores are often particularly sensitive to habitat loss and fragmentation given their large area requirements and sensitivity to human disturbance, and are frequently used as focal species to guide broad-scale landscape connectivity planning [19–24].

Many past population connectivity assessments have focused on narrow linear corridors of habitat between core populations [25–27]. Although small, narrow, linear habitat corridors can be important in some landscapes (e.g., areas with high habitat fragmentation), it is increasingly recognized that connectivity planning must be applied to broad landscapes to conserve plant and animal populations [2, 28–31]. It has been argued that the narrow focus on movement among discrete habitat patches via narrow linear corridors should be subsumed as a special case of the general process of organisms traversing resistant landscapes [32, 33], and that identifying areas where organism movement is concentrated or where it is blocked is a fundamentally important task in guiding conservation [33]. Thus, an important goal in applied movement ecology is intersecting predicted corridor networks with road networks across broad landscapes to identify and prioritize corridors and recommend where the potential impacts of roads on animals can be mitigated.

Reliable prediction of corridor networks for animals depends on knowledge of three things: (1) the distribution and abundance of the species, (2) the dispersal and movement ability of the species and (3) the pattern of differential movement cost, or resistance, across the landscape [34, 35]. Resistance models used to predict connectivity should be based on empirical data that reliably reflect the effects of landscape features on organism movement [36]. However, the vast majority of connectivity analyses have used resistance maps that are based on unvalidated expert opinion [34, 36]. Reliable predictions of population connectivity should be based on resistance surfaces that are empirically derived [37], and ideally produced through multi-step empirical modeling (sensu [36]). For example, there is particular value in estimating resistance through several methods using independent data. However, very few examples of empirically validated resistance surfaces produced with multiple methods applied to independent data exist (but see [38, 39]).

In this paper we evaluated regional connectivity for American black bear (*Ursus americanus*) populations and specifically evaluate the intersection of the network of predicted corridors with highways. Cushman et al. [40] used individual-based landscape genetics analysis to predict landscape resistance for black bear in the northwestern part of the analysis area, and found that population connectivity is facilitated by middle elevation forest and resisted by non-forest areas and roads. Subsequently, Cushman and Lewis [38] tested the validity of this resistance map using independent movement data and confirmed the relationships between landscape resistance, forest cover, human land use and roads. Further validation was conducted through process-pattern modeling with this resistance map using spatially-explicit, individual-based simulations [41, 42]. Short Bull et al. [39] evaluated the consistency of this resistance model by conducting a meta-replicated landscape genetics study in which the same candidate resistance hypotheses were tested in 12 analysis areas across the northern Rocky Mountains, and confirmed that forest cover at middle elevations facilitates movement and roads and non-forest land cover resist it across the full extent of the present analysis area.

Our goal in this paper was to use the resistance map produced and validated in these earlier studies to predict the location and strength of movement corridors across a 30.2 million hectare analysis area in Montana and Idaho, USA, and identify locations where predicted corridors intersect major highways. Our analysis is motivated by four objectives. First, we evaluated the effectiveness of factorial least cost path corridor mapping on an empirical resistance surface in reflecting the frequency of highway crossings by American black bear. Second, we predicted the location and intensity of movement corridors for American black bear. Third, we identified where these corridors cross major highways and rank the intensity of these crossings. Finally, we evaluated the optimality of placement of 34 recently created crossing structures on one stretch of highway in the analysis area for black bear movement.

## Results and discussion

### Location and intensity of movement corridors

**Figure 1:**
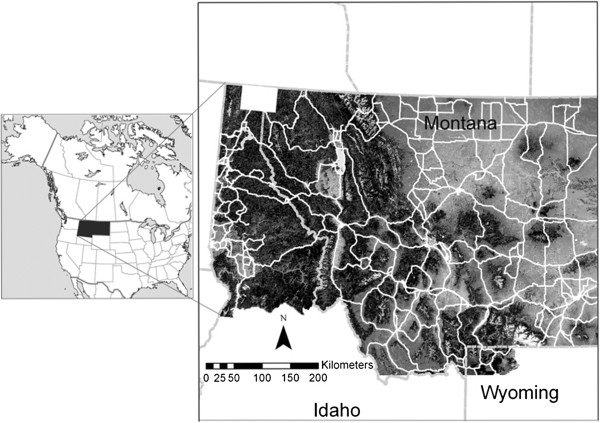
**Analysis area location and resistance map produced by empirically optimizing the correlation between genetic differentiation among individual black bears and landscape features**[40]**, and verified in a meta-replicated landscape genetic study**[39]**and by modeling the relationship between movement path selection by American black bear and landscape features**[38]**.** Dark areas are predicted to have low resistance to movement while light colored areas are predicted to represent high movement cost. State boundaries are shown in dashed grey lines, and the network of state, federal and interstate highways is shown in white lines. The white box in the upper left corner depicts the location where the highway crossing data used to validate the predicted corridors were collected.

**Figure 2:**
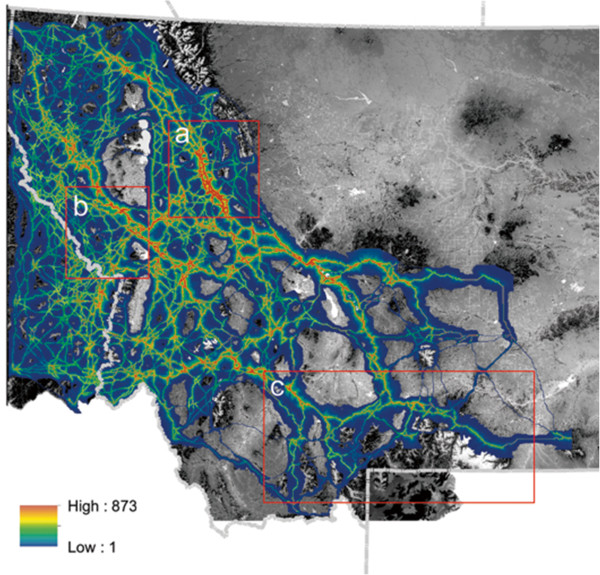
**Map of cumulative factorial least cost path network across the full extent of the analysis area.** Local corridor intensity is mapped from a minimum of 1 (dark blue) to the maximum value of 873 (burnt orange). The areas not covered by this color gradient are predicted to have no least cost corridors traversing them. Three areas are highlighted in red boxes: **(a)** Bob Marshal Wilderness Complex, **(b)** Bitterroot Mountains and Reservation Divide, **(c)** Greater Yellowstone Ecosystem.

The results are based on least cost corridor modeling on an empirically derived resistance surface (Figure 1), which reflects relative movement cost across the landscape [3, 38]. Our factorial least cost path analysis predicted the strength of movement corridors across this resistance map, producing a gradient of corridor intensity across the analysis area (Figure 2). A total of just over 30% of the 30.2 million hectare analysis area was predicted to be covered by our least cost corridor network (Table 1). The proportion of the landscape covered by different corridor intensities declined rapidly with increasing corridor intensity. For example, just over 2% of the analysis area was covered by corridors of intensity 100 or greater, and less than 1/10^th^ of 1% of the analysis area was covered by corridors of intensity greater than 500 (Table 1).

**Table 1:** **Extent of corridors of differentintesnsity and percent protected by federal management**

Corridor strength	Hectares	Percent federal
> 0	9,106,597	75.1
> 100	683,014	82.2
> 200	231,776	80.0
> 300	90,551	72.2
> 400	41,959	80.6
> 500	12,139	92.6

>0 – all predicted corridors, > 100 – predicted corridors with intensity greater than 100, >200 – predicted corridors with intensity greater than 200, >300 – predicted corridors with intensity greater than 300, >400 – predicted corridors with intensity greater than 400, >500 – predicted corridors with intensity greater than 500. Intensity is defined as the concentration of number of least cost routes predicted to traverse a given cell.

We identified major connectivity nodes through which large numbers of least cost routes pass (Figure 2). The strongest such linkage node area is in the southern Bob Marshal Wilderness area in the Swan River Valley, and represents the confluence of paths leading from all of the southern portions of the analysis area to the Northern Continental Divide Ecosystem (Figure 2a). In most of the rest of the analysis area the areas of highest corridor intensity form a dendritic network showing the major linkages among portions of the analysis area. Notable examples are the strong intensity corridor network that runs from northwest to southeast along the Bitterroot Mountains and Reservation Divide northwest of Missoula (Figure 2b), which is a confluence of all routes leading to the Idaho Panhandle from other portions of the analysis area, and the strong corridor route that leads from the southern edge of the Bob Marshall Wilderness Complex to the Greater Yellowstone Ecosystem along the Big Belt and Little Belt Mountains (Figure 2c).

The network of predicted least cost corridors also shows extensive areas of lesser corridor intensity (Figure 2). Areas where the landscape is highly permeable and in which the optimal least-cost route is only marginally more optimal than alternative routes are indicated by wide areas of lesser corridor intensity. Conversely, areas where the predicted corridor routes are narrow indicate locations in the analysis area where local landscape resistance surrounding the optimal corridor route is high, constraining available movement path choices.

**Figure 3:**
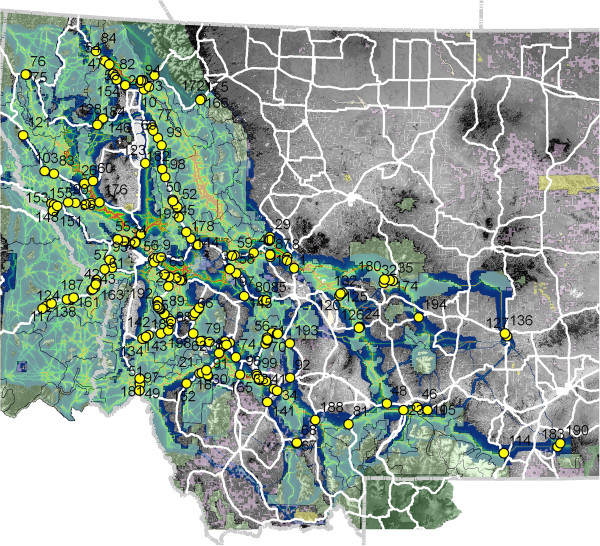
**Overlay of the cumulative factorial least cost path corridor network on the Federal ownership and highways map, with the 200 strongest corridor highway crossing locations labeled in yellow dots.** The numbers refer to the ranking of the strength of corridor-highway intersection. The extent of federal ownership is shown in transparent light green polygon overlay.

Intersection of the least cost corridor network with federally managed lands shows that most of the predicted corridor network is on public land (Table 1, Figure 3). For example, over 75% of the total predicted corridor network is on federal lands (Table 1), and the proportion increases for the portions of the corridor network of highest intensity, such as over 90% of the corridors with intensity value of 500 or greater occur on federal lands. This is particularly true in the western part of the analysis area. In the eastern and southeastern parts of the analysis area public land is less extensive and generally concentrated along north–south oriented mountain ranges. The dominant predicted corridor routes run along these ranges, but there are multiple locations where they traverse private land and low elevation, non-forested valleys.

### Effectiveness of corridor mapping in predicting highway crossings by American black bear

We evaluated the effectiveness of our corridor network in predicting highway crossing locations of black bears in northern Idaho. The corridor intensity was higher for bear crossing locations (median corridor intensity of 56 observations was 115.84, while mean and standard deviation of the medians of 1x10^6^ spatial randomizations were 83.8 and 23.6, respectively). Only 7.5% of the 1x10^6^ randomizations produced a median corridor intensity larger than that of the 56 bear highway crossing locations, indicating that corridor intensity predictions for the interpolated bear crossing locations were higher than expected based on the available crossing locations.

### Intersection of corridors and highways

We identified all locations where predicted corridor routes crossed major highways. We ranked these by corridor intensity at the crossing location, and mapped the 200 locations with the highest intensities (Figure 3). These intersections are potential barrier locations, given that they are places where potentially important movement routes intersect potentially important barriers (highways), and are clustered in the central and south-central parts of the analysis area. In contrast there are relatively few potential major corridor intersections with highways in the Northern Continental Divide, Central Idaho, and Idaho-Panhandle-Northwest Montana regions. The ranking, geographical coordinates, and corridor intensity values of these crossings are in Additional file 1.

### Evaluating potential effectiveness of existing crossing structures for black bear

**Figure 4:**
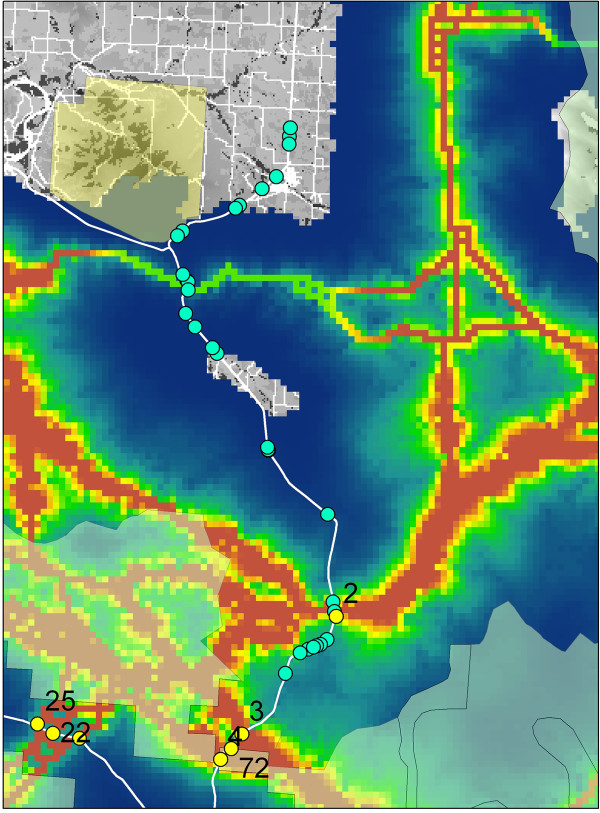
**Locations of highway overpass and underpass structures for wildlife passage installed on U.S.** Highway 93 between Evaro and Arlee Montana in comparison with predicted major corridor highway corssings. Blue dots are 34 highway crossing structures suitable for bear movement. Yellow dots are locations where strong predicted movement corridors cross highways. The numbers refer to the ranking of the strength of corridor-highway intersection.

We evaluated the potential effectiveness of a wildlife crossing structure project recently completed along U.S. 93 between Evaro Hill and Ronan, Montana, for black bears. Figure 4 shows the locations of the crossing structures relative to the predicted major corridor crossing locations. There are two crossing structures placed at the second most intense corridor location in the entire analysis area (Figure 4). Importantly, one of these structures is a major wildlife overpass bridge, which entailed the largest capital investment and was intended to be the keystone linkage in the crossing structure system. There are three crossing structures co-located with another relatively weak predicted corridor route near the junction of U.S. 93 and Hwy 200.

### Predicting synoptic regional population connectivity

Our project provides one of the first synoptic evaluations of population connectivity for a wildlife species across a regional analysis area based on an empirically developed and empirically verified resistance map. Our analysis is strengthened by its use of a resistance map that was derived from empirical optimization of genetic differentiation in relation to multiple landscape variables [40], and which was subsequently verified in a large meta-replicated landscape genetics study [39] and by a study linking movement path selection to genetic differentiation [38]. Furthermore, the analysis reported here verifies that the corridors predicted on the basis of this resistance map have some success predicting the actual locations where black bears have been recorded crossing a major federal highway in the analysis area. Locations where bears were observed crossing the highway were on average 38% higher in predicted corridor intensity than locations where they were not, and the randomization test showed modestly significant difference in the probability of crossing based on corridor intensity (p = 0.075).

Many previous regional scale connectivity approaches model movement from a small number of source patches to a small number of destination patches (e.g., [43, 44]; but see [45, 46]), which may oversimplify the characterization and distribution of habitat patches and exclude multiple core patches from the analysis. The framework we adopted incorporates additional information about how landscape resistance across broad landscapes affects regional connectivity synoptically across a vast land area, which enables the identification of key nodes of connectivity, ranking of corridor intensity, and prioritizing locations for mitigation action based on the potential severity of impacts of roads on important predicted corridors.

Cushman et al. [32] used a variation of the factorial least cost path analysis presented here to evaluate population connectivity for American black bear between the Greater Yellowstone Ecosystem and the Canadian border, and highlighted two main routes with limited width and crossing more than 20 potential highway barriers between the Greater Yellowstone Ecosystem and the Northern Continental Divide Ecosystem and Canada. That analysis, however, provided no additional information on synoptic connectivity across the analysis area, and is limited to inferences about connectivity between Yellowstone to the Canadian border, while this analysis enables predicting of connectivity from any location to any other location in the analysis area. However, boundary effects, which are inherent with broad-scale analyses, might be present and evaluating connectivity at even broader extents would provide a clearer depiction of connectivity to adjoining areas in the US and Canada.

The analysis presented here is consistent with the findings of Cushman et al. [35] who predicted the existence of narrow predicted corridors linking Greater Yellowstone to the rest of the analysis area, and also provides additional prediction of connectivity from location to location across the entire region. The results highlight two important patterns. First, the existence of major connectivity “nodes” of high corridor intensity depict the major routes predicted to be dominantly important in regional connectivity for black bear. Second, this analysis combined the use of factorial least cost path modeling to locate optimal movement routes with resistant kernels to evaluate the selectivity of these optimal routes relative to the resistance of the local landscape in which they are embedded. This allowed us to evaluate the locations of the landscape where connectivity is “funneled” along the least cost corridors due to restricted movement driven by high resistance of the surrounding landscape, and identify regions where the surrounding landscape is nearly as suitable for movement as the predicted corridor route and likely to receive nonlocalized use in movement.

The overlay of the predicted corridor connectivity network with federal land and highways indicated three important things. First, the vast majority of the corridor network is “protected” under federal management (Table 1). This is consistent with Cushman et al. [23] who found that for species, such as the American black bear, that are associated with mid-to-high elevation forest, federal lands protected the vast majority of the connectivity network. However, they also found that species associated with lower elevations and non-forest habitats were very poorly protected by the network of federal lands. Thus, our results for American black bear do not suggest high regional connectivity and high sufficiency of protected lands for species associated with different habitats. Second, the pattern of major intersections of the corridor network with highways shows that in the western and northern parts of the analysis area there are relatively few “major” corridor-highway crossings given that there are many alternative movement routes through these well connected landscapes coupled with relatively few major highways. In the central part of the analysis area there are somewhat more restricted movement corridors among more strongly defined mountain ranges separated by large valleys and a higher density of major highways, leading to the highest density of major intersections between highways and predicted movement corridors. Third, the predicted corridors connecting the Greater Yellowstone Ecosystem to the rest of the analysis area are those that also have the least complete protection by federal ownership. This makes them additionally vulnerable to habitat loss and fragmentation as the population grows and private land is developed. As previously found by Cushman et al. [32], there are a number of locations where narrow and restrictive movement corridors between the Greater Yellowstone Ecosystem and other parts of the analysis area intersect major highways.

### Evaluating the optimality of wildlife crossing structure placement

In the past several decades enormous investments have been made to install highway crossing and fencing structures to reduce wildlife-vehicle collisions while enabling wildlife to traverse the landscape [47–50]. In most cases the placement of these structures has been guided by either expert opinion or fine scale data about local animal movements in proximity to the highway [48, 51]. Few efforts have evaluated optimality of placement of crossing structures relative to broad-scale connectivity across the population. Predicting optimal placement of highway crossing structures is critical to ensure they accomplish their objectives of maintaining regional scale population connectivity.

The US Highway 93 reconstruction project in Montana is one of the most extensive wildlife-sensitive highway installations in North America [49], involving 41 fish- and wildlife crossing structures and extensive wildlife exclusion fencing. Our results indicated that several of these wildlife crossing structures were located close to some of the strongest predicted corridors in the analysis area. In particular, a major wildlife overpass bridge structure, by far the largest and most important structure installed, was located at our second highest ranked corridor-highway crossing location in the entire analysis area. While the siting of these crossing structures was not intended to be optimal for black bear movement, it is encouraging that the design seems to provide substantial connectivity for this forest generalist species.

The regional-scale connectivity map we produced and the locations and ranking of intersections with the highway network could be of utility in future efforts to design and locate wildlife highway crossing structures in other parts of the analysis area. The ranking of the 200 most intense corridor-highway intersections could be used to guide crossing structure placement and be compared to other studies that evaluated habitat selection of road crossing location at multiple spatial extents (e.g., [52]). Black bear could potentially be used as a surrogate to protect generalist forest mammals (e.g. [7]); however, recent research suggests that black bears might not act as an effective connectivity umbrella for many other species (e.g. [53]).

### Validation of predicted corridors

Corridors produced by modeling have been criticized for lacking supporting movement data [29, 30] and because they may contain errors in model parameters or incorrect assumptions [37]. Our study mitigated this by using a resistance map that was empirically optimized [40], verified in a large-scale meta-replicated study [39], tested against a large sample of movement data [38], and validated by pattern-process modeling [33, 41]. However, another key consideration is the degree to which dispersing American black bears select paths that match the predictions of our corridor network. Several field studies have evaluated the efficacy of existing corridors [54, 55] and corridors constructed as part of landscape-level experiments [56, 57]. However, formal evaluation of the performance of corridors has rarely been done at landscape scales [42]. Our analysis is one of the first to use independent movement data to validate predicted corridor networks, and verified that the intensity of our predicted corridors was related to the locations where bears crossed a major highway. Our randomization test results were moderately strong (p = 0.075 with 38% effect size), and there are several possible reasons why they were not stronger. First, the resistance map was produced by optimizing landscape resistance in relation to the genetic structure of the population and the movement data were taken from adult resident bears. Gene flow is mediated primarily by juvenile dispersal, and this may not be governed by the same factors that drive movement of adults within permanent home ranges. Cushman and Lewis [38] evaluated how well a resistance layer produced by analyzing movement pathways of resident adult black bears matched that produced by landscape genetic analysis of gene flow within this study area. They found a general agreement, with both models supporting low resistance for forest and middle elevations, and both suggesting higher resistance for non-forest, roads and high elevation areas. The models did differ in the relative resistance of these variables, however, which could partly explain the modest effect size seen in the randomization test reported here.

## Conclusions

Factorial least cost path modeling coupled with resistant kernel analysis provides detailed, synoptic information about connectivity across populations that vary in distribution and density in complex landscapes. We identified several “linkage nodes” where a large number of least cost routes were funneled through the same cells. These are predicted to be the dominant routes for movement and gene flow of black bear across the analysis area. Conversely, we identified a number of areas where there were predicted to be no movement paths. These are predicted to be barriers for black bear movement. The vast majority of the predicted corridor network was “protected” under federal management. However, narrow, linear corridors connecting the Greater Yellowstone Ecosystem to the rest of the analysis area had limited protection by Federal ownership, making them particularly vulnerable to habitat loss and fragmentation. The ranking of intensity of corridor-highway intersections (Additional file 1) may be of use in prioritizing locations for conservation or mitigation, and in optimizing the siting of projects aimed at enhancing permeability of highways to black bear dispersal. Specifically, our results could be used to quantify the structure of the connectivity network, identify critical linkage nodes and core areas, map potential barriers and fracture zones, and prioritize locations for mitigation, restoration and conservation actions.

## Methods

### Analysis area

The analysis area includes Montana and northern Idaho in the United States Rocky Mountains (Figure 1). The analysis area contains large areas of federally owned land, including U.S. Forest Service, National Park Service, U.S. Fish and Wildlife Service and Bureau of Land Management. The analysis area also includes extensive private land, mainly in the large valleys which lie between major mountain ranges. The human population in the analysis area is growing more rapidly than most areas of the United States (U.S. Census Data), and is concentrated in these valley locations. In addition, an extensive network of Federal, State and Interstate highways traverses the analysis area, potentially impeding animal movement.

### Resistance map

The genetic characteristics of individuals sampled across landscapes allows one to identify population units, localize genetic barriers, and quantify the influence of landscape features on gene flow [58, 59]. Cushman et al. [40] predicted landscape resistance to black bear gene flow in a sub-region of the analysis area using a multi-model least cost-path analysis based on molecular genetics. Their analysis identified forest cover and elevation as major factors affecting gene flow, with gene flow facilitated by closed canopy forest at middle elevations and resisted by non-forest and extremely high elevations (Figure 1). Subsequently, Short Bull et al. [39] evaluated the generality of the Cushman et al. [40] resistance map by evaluating the same suite of hypotheses regarding landscape effects on gene flow in eleven additional analysis areas distributed across Montana. They confirmed that elevation, forest cover and roads had strong influences on gene flow, supporting the generality of the Cushman et al. [40] resistance model.

The strongest inferences about landscape resistance to movement and gene flow are yielded by studies in which several methods and datasets yield convergent results. Cushman and Lewis [38] utilized this approach to evaluate the degree to which movement behavior of individual black bears explained the pattern of genetic differentiation described in Cushman et al. [40]. Their study confirmed that black bear movement path selection was driven by elevation, forest cover, roads and human land use, consistent with the landscape resistance model inferred from landscape genetic analyses by Cushman et al. [40]. In this study, we apply the Cushman et al. [40] resistance model across the full extent of the analysis area and use it as a base for factorial least cost path analyses.

### Least cost path analysis

We predicted the extent and strength of the corridor network for black bears across the analysis area with a combination of factorial least cost path and resistant kernel analysis, using the UNICOR software [60]. The factorial least cost path analysis maps the least cost routes among all combinations of source locations distributed at 5 km spacing across all forested areas in the analysis area, amounting to a total of 3,837 points. UNICOR uses Dijkstra’s algorithm [61] to solve the single source shortest path problem from every mapped source location on a landscape to every other source location [60], producing predicted least-cost path routes from each source point to each destination point (7,359,366 individual least cost paths).

These least cost paths are single pixel in width, and record the route of a least cost path from the source to the destination pixel. Animals utilizing real landscapes are unlikely to know and use these narrow optimal paths. To more realistically represent the probability of utilization of each location in the landscape as a movement path we used a cost-kernel to predict relative cost of using pixels in the vicinity of the least cost path. This was accomplished in two steps. First, we produced the cumulative least cost path network by summing all individual least cost paths among individuals (e.g., [32]). The value of this summation is corridor intensity, and equals the number of least cost paths that pass through any given pixel. Second, we calculated resistant kernels [33, 62] from each cell containing a link in the least-cost path network, with the volume of the kernel proportional to the value of the summed cumulative least cost path network at that location. Resistant kernels predict the extent and shape of the area reachable by dispersal from a source, based on a least-cost hull on a resistance surface. By placing these on every pixel within the factorial least cost path network and scaling the volume proportional to the corridor intensity at each pixel in the network, the resistant kernels produce a map of predicted corridors that are not restricted to single pixel least cost paths, but smoothed as a function of cumulative cost and corridor intensity. The kernels were calculated in UNICOR with a 10,000 m maximum dispersal ability in ideal habitat (cells with resistance 1 in the resistance map).

The analysis produced a map of corridor intensity in which cell values reflect the number of least cost paths among the network of all least cost paths connecting the 3,837 source points that traverse that pixel, after kernel smoothing. This corridor intensity value represents predicted relative frequency of expected use of the corridor, assuming bears move across the landscape following low cost routes on the resistance map. It provides a more realistic depiction of the corridor network, with areas of extensive relatively low resistance habitat predicted to have wider and less intense corridors, while areas that are constrained in narrow passages between high resistance features are mapped as more narrow and high intensity corridors. This approach is an improvement over past approaches that assume use of the least cost path or smooth the least cost paths with uniform kernels regardless of context (e.g., [33]). Importantly, our approach reflects that actual paths taken by bears will imperfectly follow least cost routes due to stochastic behavioral choices of individual animals, lack of perfect knowledge of the surrounding environment, and the pattern of relative cost of different local movement choices across the landscape.

### Validating predicted corridors

The corridor network produced in this analysis is based on least cost paths across a resistance surface that was derived from empirical analysis of gene flow across multiple analysis areas and path-level analysis of bear movement behavior. However, the goal of this paper is to predict bear movement, in particular highway crossings. As such it is important to further validate the corridor predictions with observations of actual bear highway crossing locations. We use movement data obtained by Lewis et al. [52] to test the congruence of our predicted corridor network with the locations of actual bear highway crossing events. Specifically, bears were trapped from June to mid-August in 2004–2006 in the Purcell Mountain range of northern Idaho using Aldrich foot snares [63] and fitted with Lotek 3300 L GPS collars. Collars were programmed to record a location every 20 min from April (den emergence) to November (den entrance) and information was stored on the collar, providing consistent fine scale movement data with high fix rates and small location errors [52]. This movement data set provided 56 instances where collared bears crossed US Highway 95 in northern Idaho.

We used these instances of highway crossing as a dependent variable set to test the null hypothesis that the observed bear crossings do not depend on the connectivity score of the landscape, by comparing the 56 observed crossings to the distribution of 56 randomly sampled crossings. We sampled corridor intensity along the entire length of highway 95 within the analysis area at 30 m intervals. Corridor intensity is the value of the corridor grid at that location, and reflects the number of least cost paths among source locations that pass through that location. Heuristically, the intensity of the corridor grid reflects the relative probability of use of that location of the network. We evaluated the association between actual bear crossings and the predicted corridor network through a spatial randomization test. The dependent variable in this randomization test is the median value of corridor intensity for the 56 actual crossing locations. The randomization test conducts a random draw, without replacement, of 56 locations from the population of all locations along Highway 95 within the analysis area, and calculates the median corridor intensity of this random sample of potential highway crossing sites. We repeated this 1x10^6^ times, and then compared the median corridor intensity of the 56 actual highway crossings, to the distribution of 1x10^6^ median corridor intensities of available highway crossings produced through the randomization. Spatial randomization testing of this kind is recommended in cases, such as this, where there is spatial dependence among observations, and produces an unbiased estimate of the probability of the observed outcome given the data [64].

### Intersecting corridors with highways

One of our primary goals was to identify where predicted corridors cross major highways and rank the intensity of these crossings across the full 30.2 million hectare analysis area. To accomplish this objective we intersected state, federal and interstate highways with our predicted corridor network. We ranked these intersections based on the intensity of the corridor, as defined above, at the site of its intersection with the highway. We mapped the 200 intersections of the corridor network with highways that had the highest corridor intensity.

### Evaluating the optimality of a wildlife highway crossing structure project

The US Highway 93 (US 93) reconstruction project installed 41 fish- and wildlife crossing structures and approximately 26.7 km of wildlife exclusion fencing. The mitigation measures were aimed at improving safety for the traveling public through reducing wildlife-vehicle collisions and allowing wildlife to continue to move across the landscape and the road. Both the road length and number of wildlife crossing structures in the US 93 project make it the most extensive mitigation project of its kind in North America to date [49]. Thirty-four of the 41 crossing structures are deemed suitable for usage by bears (M. Sawaya pers. comm.). We evaluated the location of these relative to our predicted movement corridors to assess the optimality of placement relative to the regional-scale corridor network.

## Availability of supporting data

The data sets supporting the results of this article are available in the University of Montana Computational Ecology Laboratory repository, [unique persistent identifier and hyperlink to dataset(s) in http://format].

## Electronic supplementary material

Additional file 1: **Listing of the 200 most intense intersections of the predicted corridor network and highways, as ranked by the intensity value of the corridor network at the location of the highway intersection.** X and Y are coordinates of the crossing in an Albers conformal conic projection. Latitude and Longitude are coordinates in decimal degrees. Datum is NAD83. “Corridor” is the corridor network intensity at the location of the highway intersection. Parameters of the Albers projection: False_Easting: 600000; False_Northing: 0; Central_Meridian: -109.5; Standard_Parallel_1: 46.0; Standard_Parallel_2: 48.0; Latitude_Of_Origin: 44.0. (DOCX 31 KB)
