# Supplementary material for: Evaluating the intersection of a regional wildlife connectivity network with highways
Source: Mov Ecol. 2013 Nov 22;1(1):12. doi: 10.1186/2051-3933-1-12 (PMC4337767; doi:10.1186/2051-3933-1-12)
Supplement: Supplementary file 1 — Additional file 1: Listing of the 200 most intense intersections of the predicted corridor network and highways, as ranked by the intensity value of the corridor network at the location of the highway intersection. X and Y are coordinates of the crossing in an Albers conformal conic projection. Latitude and Longitude are coordinates in decimal degrees. Datum is NAD83. “Corridor” is the corridor network intensity at the location of the highway intersection. Parameters of the Albers projection: False_Easting: 600000; False_Northing: 0; Central_Meridian: -109.5; Standard_Parallel_1: 46.0; Standard_Parallel_2: 48.0; Latitude_Of_Origin: 44.0. (DOCX 31 KB) [file 40462_2013_11_MOESM1_ESM.docx]

Additional file 1. Listing of the 200 most intense intersections of the predicted corridor network and highways, as ranked by the intensity value of the corridor network at the location of the highway intersection. X and Y are coordinates of the crossing in an Albers conformal conic projection. Latitude and Longitude are coordinates in decimal degrees. Datum is NAD83. “Corridor” is the corridor network intensity at the location of the highway intersection. Parameters of the Albers projection: False_Easting: 600000; False_Northing: 0; Central_Meridian: -109.5; Standard_Parallel_1: 46.0; Standard_Parallel_2: 48.0; Latitude_Of_Origin: 44.0.

| Crossing Number | x | y | Latitude | Longitude | Corridor |
| --- | --- | --- | --- | --- | --- |
| 1 | 407504 | 319901 | 46.85134 | -112.033 | 872.1434 |
| 2 | 255584 | 353381 | 47.0906 | -114.048 | 516 |
| 3 | 250064 | 346481 | 47.02575 | -114.115 | 486.6094 |
| 4 | 248834 | 345011 | 47.0119 | -114.13 | 484.5469 |
| 5 | 267584 | 328601 | 46.87422 | -113.872 | 479.6163 |
| 6 | 269264 | 329801 | 46.88583 | -113.851 | 479.6162 |
| 7 | 268664 | 325601 | 46.84782 | -113.855 | 479.1352 |
| 8 | 400664 | 330701 | 46.9464 | -112.128 | 477.6155 |
| 9 | 272324 | 330161 | 46.89058 | -113.811 | 473.7218 |
| 10 | 258464 | 497201 | 48.38336 | -114.121 | 445.6433 |
| 11 | 256304 | 505841 | 48.45979 | -114.157 | 434.8338 |
| 12 | 257384 | 506561 | 48.46683 | -114.143 | 423.3334 |
| 13 | 296384 | 308081 | 46.70369 | -113.481 | 405.0283 |
| 14 | 294224 | 308321 | 46.70486 | -113.509 | 395.2233 |
| 15 | 205544 | 384881 | 47.34532 | -114.733 | 387.0723 |
| 16 | 373124 | 208841 | 45.84215 | -112.429 | 375.8005 |
| 17 | 384104 | 333761 | 46.96872 | -112.346 | 373.6699 |
| 18 | 320024 | 216941 | 45.89507 | -113.117 | 365.5156 |
| 19 | 240584 | 346241 | 47.01852 | -114.239 | 357.8479 |
| 20 | 263504 | 499901 | 48.41025 | -114.055 | 357.5589 |
| 21 | 313544 | 215501 | 45.87941 | -113.2 | 356.9164 |
| 22 | 239024 | 346541 | 47.02036 | -114.26 | 355.2083 |
| 23 | 516224 | 179141 | 45.6078 | -110.582 | 347.7218 |
| 24 | 472664 | 261761 | 46.3442 | -111.163 | 343.7182 |
| 25 | 238124 | 347081 | 47.02472 | -114.272 | 339.2288 |
| 26 | 199424 | 404141 | 47.51444 | -114.831 | 331.7585 |
| 27 | 290504 | 310361 | 46.72146 | -113.559 | 325.7656 |
| 28 | 382544 | 347201 | 47.08901 | -112.373 | 322.8447 |
| 29 | 383744 | 350861 | 47.1223 | -112.359 | 318.6497 |
| 30 | 321392 | 214889 | 45.87719 | -113.098 | 315.2086 |
| 31 | 283304 | 311441 | 46.72778 | -113.654 | 312.8963 |
| 32 | 496424 | 305141 | 46.73843 | -110.864 | 311.6481 |
| 33 | 387344 | 200021 | 45.76746 | -112.242 | 301.7586 |
| 34 | 390584 | 198221 | 45.75228 | -112.2 | 301.7586 |
| 35 | 505424 | 307481 | 46.76081 | -110.747 | 295.3361 |
| 36 | 385544 | 256001 | 46.27021 | -112.291 | 291.7368 |
| 37 | 343064 | 319541 | 46.82631 | -112.877 | 289.2283 |
| 38 | 390584 | 254741 | 46.26048 | -112.225 | 287.4128 |
| 39 | 227864 | 512861 | 48.507 | -114.547 | 287.0926 |
| 40 | 367424 | 335321 | 46.9771 | -112.566 | 285.7714 |
| 41 | 379784 | 207761 | 45.83464 | -112.343 | 284.5922 |
| 42 | 209864 | 301361 | 46.59834 | -114.604 | 280.1644 |
| 43 | 210224 | 302801 | 46.61147 | -114.601 | 279.8401 |
| 44 | 391424 | 254801 | 46.26128 | -112.214 | 277.1921 |
| 45 | 289064 | 383261 | 47.37554 | -113.628 | 274.2837 |
| 46 | 529364 | 182201 | 45.63683 | -110.414 | 273.6025 |
| 47 | 223304 | 522881 | 48.59429 | -114.617 | 273.4606 |
| 48 | 499484 | 185441 | 45.66221 | -110.798 | 271.9389 |
| 49 | 254144 | 205781 | 45.76432 | -113.956 | 269.7161 |
| 50 | 286724 | 387761 | 47.41485 | -113.662 | 267.3669 |
| 51 | 254504 | 204701 | 45.7548 | -113.951 | 266.8565 |
| 52 | 291224 | 380561 | 47.35231 | -113.598 | 261.0213 |
| 53 | 221924 | 523841 | 48.60209 | -114.637 | 260.8736 |
| 54 | 219152 | 525929 | 48.6192 | -114.676 | 259.2778 |
| 55 | 232364 | 348521 | 47.03447 | -114.349 | 258.9576 |
| 56 | 380264 | 250001 | 46.21457 | -112.357 | 257.6346 |
| 57 | 226964 | 320441 | 46.7794 | -114.397 | 246.7691 |
| 58 | 348104 | 332861 | 46.94794 | -112.819 | 245.9472 |
| 59 | 345944 | 332141 | 46.94064 | -112.847 | 245.862 |
| 60 | 207704 | 406481 | 47.54044 | -114.724 | 243.4651 |
| 61 | 219464 | 318761 | 46.76007 | -114.494 | 241.977 |
| 62 | 307244 | 255281 | 46.23417 | -113.305 | 240.7496 |
| 63 | 278984 | 313781 | 46.74673 | -113.712 | 233.6947 |
| 64 | 380401 | 246384 | 46.18209 | -112.353 | 227.5772 |
| 65 | 282584 | 270221 | 46.35719 | -113.635 | 220.0552 |
| 66 | 307064 | 272741 | 46.39097 | -113.319 | 219.9774 |
| 67 | 409844 | 145841 | 45.28682 | -111.932 | 219.5063 |
| 68 | 410024 | 146561 | 45.29334 | -111.93 | 218.5438 |
| 69 | 272324 | 449321 | 47.9606 | -113.898 | 218.0109 |
| 70 | 379604 | 245201 | 46.1712 | -112.363 | 210.8326 |
| 71 | 379064 | 244841 | 46.16779 | -112.37 | 208.0073 |
| 72 | 249464 | 345641 | 47.01789 | -114.122 | 206.3551 |
| 73 | 257024 | 506201 | 48.4634 | -114.148 | 201.4308 |
| 74 | 349544 | 231701 | 46.03933 | -112.745 | 197.9924 |
| 75 | 142184 | 512321 | 48.44697 | -115.702 | 195.8257 |
| 76 | 140924 | 512321 | 48.44607 | -115.719 | 195.7682 |
| 77 | 266744 | 459581 | 48.04989 | -113.981 | 193.0446 |
| 78 | 316784 | 247721 | 46.17033 | -113.177 | 184.094 |
| 79 | 314624 | 247361 | 46.16618 | -113.205 | 184.0607 |
| 80 | 376544 | 288221 | 46.5569 | -112.423 | 183.8537 |
| 81 | 461504 | 165101 | 45.47254 | -111.279 | 183.8529 |
| 82 | 229664 | 509621 | 48.47896 | -114.52 | 179.8066 |
| 83 | 169184 | 413681 | 47.58086 | -115.241 | 179.7265 |
| 84 | 210944 | 535361 | 48.69893 | -114.796 | 179.3381 |
| 85 | 379784 | 288221 | 46.55798 | -112.381 | 178.6158 |
| 86 | 190424 | 384881 | 47.33609 | -114.933 | 177.7571 |
| 87 | 384104 | 331061 | 46.94446 | -112.345 | 177.6837 |
| 88 | 285464 | 267521 | 46.33429 | -113.596 | 175.8529 |
| 89 | 278624 | 274541 | 46.3941 | -113.689 | 175.1436 |
| 90 | 183224 | 385421 | 47.33642 | -115.028 | 174.9855 |
| 91 | 322184 | 212621 | 45.85713 | -113.087 | 174.5853 |
| 92 | 403544 | 211181 | 45.87271 | -112.039 | 171.8625 |
| 93 | 276104 | 441941 | 47.89622 | -113.842 | 169.0226 |
| 94 | 254864 | 503861 | 48.44124 | -114.175 | 163.2833 |
| 95 | 224264 | 328181 | 46.84735 | -114.439 | 162.5167 |
| 96 | 370064 | 214781 | 45.89452 | -112.472 | 158.6819 |
| 97 | 253784 | 198581 | 45.69946 | -113.956 | 158.0377 |
| 98 | 279704 | 410261 | 47.61352 | -113.771 | 157.724 |
| 99 | 370496 | 212873 | 45.87752 | -112.465 | 155.3853 |
| 100 | 337484 | 241961 | 46.12696 | -112.906 | 154.9979 |
| 101 | 310664 | 343121 | 47.02483 | -113.316 | 149.7018 |
| 102 | 312104 | 342941 | 47.02384 | -113.297 | 149.6904 |
| 103 | 159824 | 416381 | 47.59885 | -115.368 | 149.0471 |
| 104 | 160904 | 282101 | 46.39515 | -115.223 | 145.8824 |
| 105 | 539984 | 179141 | 45.61032 | -110.278 | 145.882 |
| 106 | 274664 | 279221 | 46.43423 | -113.744 | 145.8162 |
| 107 | 343424 | 245741 | 46.16323 | -112.831 | 145.776 |
| 108 | 338744 | 247181 | 46.17437 | -112.893 | 145.7549 |
| 109 | 315884 | 216041 | 45.88525 | -113.17 | 145.7032 |
| 110 | 206264 | 298121 | 46.56714 | -114.648 | 143.6598 |
| 111 | 338024 | 243041 | 46.13688 | -112.9 | 143.5856 |
| 112 | 231284 | 506921 | 48.45565 | -114.496 | 142.4924 |
| 113 | 293384 | 370661 | 47.26441 | -113.562 | 142.3717 |
| 114 | 615584 | 136301 | 45.22724 | -109.31 | 141.8865 |
| 115 | 340184 | 243761 | 46.14419 | -112.872 | 141.639 |
| 116 | 211304 | 308201 | 46.66057 | -114.591 | 141.5356 |
| 117 | 166664 | 284981 | 46.42474 | -115.151 | 141.3784 |
| 118 | 272144 | 286601 | 46.49928 | -113.782 | 140.0665 |
| 119 | 271784 | 286061 | 46.49426 | -113.786 | 139.9854 |
| 120 | 455384 | 295601 | 46.64499 | -111.398 | 139.7308 |
| 121 | 138404 | 451841 | 47.90203 | -115.689 | 133.5024 |
| 122 | 380504 | 250961 | 46.22328 | -112.354 | 132.752 |
| 123 | 259904 | 424301 | 47.72959 | -114.045 | 132.6262 |
| 124 | 157664 | 279941 | 46.37364 | -115.263 | 132.0595 |
| 125 | 452444 | 292601 | 46.61737 | -111.435 | 131.6447 |
| 126 | 471704 | 260921 | 46.33646 | -111.175 | 130.533 |
| 127 | 618824 | 252581 | 46.27345 | -109.264 | 130.1158 |
| 128 | 214184 | 464981 | 48.06926 | -114.689 | 128.2739 |
| 129 | 186104 | 288941 | 46.47258 | -114.902 | 125.8703 |
| 130 | 350264 | 314861 | 46.787 | -112.78 | 124.626 |
| 131 | 262784 | 253121 | 46.19393 | -113.879 | 123.8468 |
| 132 | 453728 | 294737 | 46.63686 | -111.419 | 123.6247 |
| 133 | 185384 | 289301 | 46.47536 | -114.912 | 123.495 |
| 134 | 256904 | 250001 | 46.16294 | -113.953 | 122.3579 |
| 135 | 209504 | 300641 | 46.59166 | -114.608 | 120.2007 |
| 136 | 616664 | 255281 | 46.29779 | -109.292 | 118.0801 |
| 137 | 380864 | 248261 | 46.19912 | -112.348 | 117.7769 |
| 138 | 181964 | 289121 | 46.47162 | -114.956 | 117.6869 |
| 139 | 372584 | 209921 | 45.85168 | -112.437 | 115.7044 |
| 140 | 321824 | 213701 | 45.86669 | -113.092 | 114.1778 |
| 141 | 380864 | 187061 | 45.64886 | -112.32 | 111.9105 |
| 142 | 263144 | 253481 | 46.19735 | -113.875 | 111.6674 |
| 143 | 275204 | 255281 | 46.21945 | -113.72 | 107.1766 |
| 144 | 304904 | 349421 | 47.07888 | -113.396 | 106.0364 |
| 145 | 357824 | 292181 | 46.58597 | -112.669 | 105.9153 |
| 146 | 217784 | 468581 | 48.1037 | -114.644 | 105.4641 |
| 147 | 166484 | 384161 | 47.31431 | -115.248 | 104.078 |
| 148 | 169064 | 383141 | 47.30685 | -115.213 | 103.9353 |
| 149 | 268904 | 511421 | 48.51649 | -113.992 | 102.7618 |
| 150 | 211304 | 305321 | 46.63472 | -114.589 | 102.0087 |
| 151 | 169544 | 377681 | 47.25819 | -115.201 | 101.908 |
| 152 | 300584 | 205421 | 45.78321 | -113.36 | 101.7614 |
| 153 | 168104 | 377141 | 47.2524 | -115.22 | 101.2918 |
| 154 | 239864 | 501401 | 48.41095 | -114.375 | 101.0886 |
| 155 | 172424 | 381641 | 47.29558 | -115.167 | 99.60808 |
| 156 | 383384 | 348161 | 47.09791 | -112.363 | 92.07676 |
| 157 | 384104 | 333041 | 46.96225 | -112.346 | 91.76727 |
| 158 | 311924 | 278681 | 46.44645 | -113.259 | 90.48135 |
| 159 | 383744 | 333401 | 46.96537 | -112.351 | 81.39253 |
| 160 | 400484 | 327281 | 46.91561 | -112.128 | 75.29225 |
| 161 | 207524 | 298841 | 46.57435 | -114.632 | 60.14894 |
| 162 | 267824 | 456521 | 48.02297 | -113.964 | 59.59926 |
| 163 | 210584 | 303881 | 46.62138 | -114.597 | 59.22805 |
| 164 | 272504 | 281561 | 46.45419 | -113.774 | 57.578 |
| 165 | 353864 | 214241 | 45.88397 | -112.68 | 56.28044 |
| 166 | 314624 | 487481 | 48.32333 | -113.358 | 53.24142 |
| 167 | 272324 | 287321 | 46.50584 | -113.78 | 49.23742 |
| 168 | 273944 | 280121 | 46.44196 | -113.754 | 48.60975 |
| 169 | 223904 | 522401 | 48.59033 | -114.609 | 48.52701 |
| 170 | 323264 | 246641 | 46.16332 | -113.093 | 47.654 |
| 171 | 320204 | 218921 | 45.91294 | -113.116 | 47.34975 |
| 172 | 315524 | 488201 | 48.33019 | -113.346 | 45.69557 |
| 173 | 255404 | 506201 | 48.46254 | -114.17 | 45.08544 |
| 174 | 502544 | 308201 | 46.76687 | -110.784 | 43.9157 |
| 175 | 314264 | 487121 | 48.31993 | -113.362 | 41.44424 |
| 176 | 214364 | 385241 | 47.35378 | -114.617 | 37.50052 |
| 177 | 273584 | 280481 | 46.44502 | -113.759 | 36.96174 |
| 178 | 300404 | 355721 | 47.13344 | -113.46 | 36.63869 |
| 179 | 309044 | 275441 | 46.4161 | -113.295 | 36.57245 |
| 180 | 496964 | 308921 | 46.7725 | -110.858 | 36.04775 |
| 181 | 254144 | 210281 | 45.80475 | -113.96 | 33.76791 |
| 182 | 276464 | 418361 | 47.68466 | -113.82 | 32.47401 |
| 183 | 669044 | 142601 | 45.28064 | -108.629 | 31.87802 |
| 184 | 212024 | 462281 | 48.04375 | -114.715 | 30.9948 |
| 185 | 380864 | 249161 | 46.20721 | -112.349 | 30.7242 |
| 186 | 260444 | 251681 | 46.17982 | -113.909 | 30.39309 |
| 187 | 188444 | 290561 | 46.48856 | -114.873 | 30.007 |
| 188 | 428564 | 169241 | 45.50228 | -111.702 | 29.95535 |
| 189 | 200324 | 383441 | 47.32925 | -114.801 | 29.67476 |
| 190 | 671924 | 146201 | 45.31274 | -108.591 | 29.4247 |
| 191 | 275564 | 330881 | 46.89864 | -113.769 | 29.32223 |
| 192 | 272144 | 281921 | 46.45725 | -113.779 | 29.11364 |
| 193 | 403364 | 245201 | 46.17857 | -112.056 | 28.49656 |
| 194 | 531164 | 271121 | 46.43695 | -110.404 | 28.09141 |
| 195 | 287144 | 386561 | 47.40427 | -113.656 | 27.626 |
| 196 | 282584 | 301721 | 46.64013 | -113.657 | 27.60224 |
| 197 | 343604 | 319001 | 46.82167 | -112.87 | 27.45509 |
| 198 | 282404 | 258161 | 46.24876 | -113.629 | 27.1076 |
| 199 | 332804 | 235481 | 46.06688 | -112.963 | 26.40166 |
| 200 | 313184 | 280841 | 46.4664 | -113.244 | 25.95047 |
